# An Analog-Front ROIC with On-Chip Non-Uniformity Compensation for Diode-Based Infrared Image Sensors

**DOI:** 10.3390/s19020298

**Published:** 2019-01-13

**Authors:** Mao Ye, Gongyuan Zhao, Yao Li, Yiqiang Zhao

**Affiliations:** 1 Tianjin Key Laboratory of Imaging and Sensing Microelectronic Technology, Tianjin University, Tianjin 300072, China; liyao@tju.edu.cn (Y.L.); yq_zhao@tju.edu.cn (Y.Z.); 2 Tianjin Infrared Imaging Technology Engineering Center, Tianjin University, Tianjin 300072, China

**Keywords:** ROIC, diode-based infrared image sensor, on-chip non-uniformity compensation, differential integrato, low power

## Abstract

This paper proposes a CMOS front-end readout-integrated circuit (ROIC) with on-chip non-uniformity compensation technique for a diode-based uncooled infrared image sensor. Two techniques are adopted to achieve on-chip non-uniformity compensation: a reference dummy metal line is introduced to alleviate the dominant non-uniformity with IR-drop presented in large pixel array, and a current splitting architecture-based variable current source for diode bias is proposed to compensate other residual non-uniformity. A differential integrator is chosen as the main amplifier of readout circuit for its superior noise performance. For low power design, a pulse-powered row buffer is designed in this work. The proposed ROIC for 384 × 288 diode-based detector array is fabricated with a 0.35-μm CMOS process. It occupies an area of 4.4 mm × 15 mm, and the power consumption is 180 mW. The measured result shows that with the proposed on-chip non-uniformity compensation, the output voltage variation is greatly reduced from 2.5 V to 60 mV.

## 1. Introduction

Compared with narrow bandgap photon-based detectors, uncooled thermal-based infrared sensors without cryogenic cooling equipment are getting popular, and expanding their application in many civil fields, because they provide the advantages of lower weight, lower cost and system complexity [1,2,3,4]. In an un-cooled infrared sensor, the detector absorbs infrared radiation, and transfers it into the heat, which changes the specific temperature dependent electrical parameters. With the development of microelectromechanical system (MEMS) technology and innovative CMOS circuit design, microbolometer and diode-based uncooled infrared sensors have emerged as research hotspot [5,6,7,8,9,10].

A microbolometer senses the thermal change of target with its temperature dependent resistance. Thin film vanadium oxide (V${}_{ox}$) and amorphous silicon (α-Si) are two types of high TCR (Temperature Coefficient of Resistance) materials that have been extensively used, but the drawbacks are also obvious; extra masks and post-CMOS fabrication steps are needed to deposit these materials and thermally isolate the pixel, and the resistor in microbolometer, which usually ranges from a hundred kΩs to several MΩs, introduces large amount of noise [11].

The temperature characteristic of a diode can be exploited as an infrared sensor. Biased with constant current, the forward voltage across the diode, V${}_{f}$, would change with temperature. Compared with microbolometers, a diode-based infrared sensor provides three advantages: firstly, diode-based sensors have the advantage of CMOS process compatibility; secondly, a diode is often fabricated with monocrystalline silicon, which presents low noise characteristic; lastly, the temperature dependency of forward voltage, dV${}_{f}$/dT, shows better uniformity than microbolometer [12].

However, there is a hidden problem which prevents a diode-based sensor from realizing its full potential. The relative variation of electrical parameter in a diode is much smaller than that in a microbolometer. Assuming the typical values of V${}_{f}$ and dV${}_{f}$/dT are 0.8 V and 1.25 mV/K, respectively, the relative variation of forward voltage, dV${}_{f}$/(V${}_{f}$dT), is only 0.156%/K, while the relative variation of resistance is as large as 2%/K. Lower relative temperature sensitivity means smaller signal voltage, and non-uniformity-induced voltage variation possibly compromises dynamic range or even saturates the readout circuit. In such an extreme case, non-uniformity correction algorithm in digital domain will also fail to improve image quality. In the presence of non-uniformity, there exists tradeoff between voltage gain and dynamic range. As is demonstrated in [12,13], voltage gain around 14 *v*/*v* is designed. As the front end of readout channel, lower voltage gain requires ADC (Analog-to-Digital Converter) with higher SNR (Signal-to-Noise Ratio) in later stages, which complicates system design and raises cost. Non-uniformity introduces the same dynamic range problem in microbolometer that needs to be handled properly too [14].

In order to improve system dynamic range performance, some readout topologies have been proposed for photon-based sensors. By adjusting the integration time with a smart reset control method and automatically switching between two capacitors with different values [15,16], dynamic range has been considerably enlarged, but these methods are ineffective in handling non-uniformity-induced dynamic range loss problem, for that non-uniformity have been mixed with useful signals.

Therefore, the fundamental way to solve this problem is to compensate non-uniformity sources. The sources of non-uniformity come from IR-drop along the common routing bus in a pixel array, mismatches of detectors themselves and current sources, offset of readout channel and so on. Some methods have been utilized to compensate for non-uniformities. In a diode-based sensor, reference pixels and dummy metal line are two classic techniques to calibrate the IR-drop, but other non-uniformity factors are often left un-compensated [17,18]. In a microbolometer, these similar residual non-uniformities are calibrated by applying corrected bias voltage with the help of sub-DAC (Digital-to-Analog Converter) [19,20,21,22].

In this work, we propose a hybrid non-uniformity-compensated ROIC for diode-based uncooled infrared sensors. A dummy metal line is introduced to alleviate the dominant IR-drop first, and a novel current splitting DAC is utilized as pixel bias to achieve residual non-uniformity compensation. Besides, a low power design technique is designed for row buffer to significantly reduce the power consumption. In Section 2, architecture and circuit details of ROIC are presented and analyzed, as well as the proposed compensation method. Experimental results and conclusions are given in Section 3 and Section 4, respectively.

## 2. ROIC Design

### 2.1. Architecture

**Figure 1:**
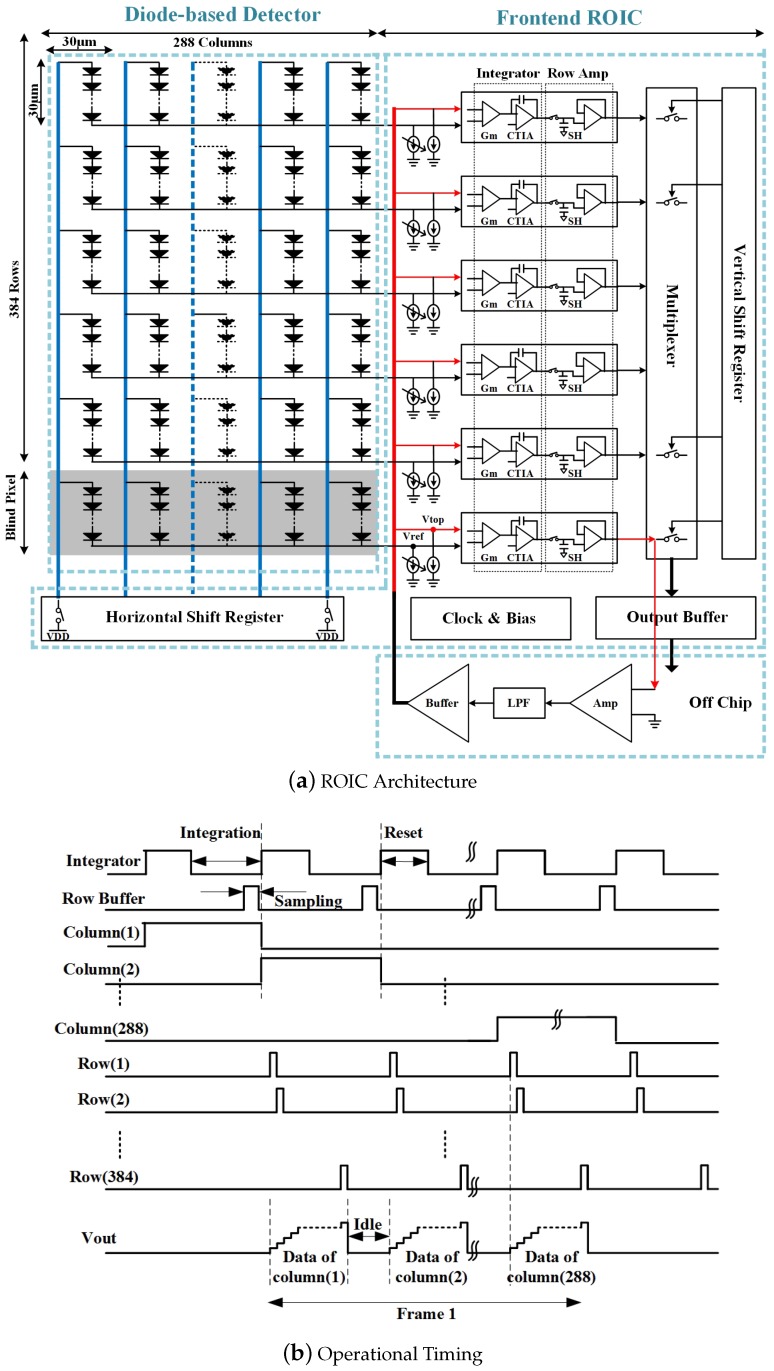
Simplified architecture (**a**) and timing diagram (**b**) of the proposed 384 × 288-frontend ROIC.

Figure 1 shows an architecture and timing diagram of the proposed ROIC. Although a diode-based uncooled infrared sensor has the potential advantage of CMOS process compatibility, detector and ROIC are fabricated separately, and integrated with each other with flip-chip technology for this demonstration prototype.

The infrared focal plane array (IRFPA) has 384 rows and 288 columns of pixels, and the pixel size is 30 μm × 30 μm. To improve temperature sensitivity, each pixel is consists of 6 series connected diodes manufactured with MEMS technology, which is forward biased by a current source shared by all pixels in a single row.

IRFPA is operated in column rolling shutter mode. When one specific column switch closes under the control of horizontal shift register, output voltages of 384 diodes in this column are processed by row channels. In each channel, an integrator-based amplifier is chosen as the first stage for its superior noise performance. In this first stage, the diode output voltage is transformed into a signal current which flows through integrating capacitor. Then, the amplified voltage is sampled, and multiplexed to common bus with pulse powered row buffer. With this sampling operation, the integrate-while-read mode is possible. Finally, voltages are read out off the chip sequentially.

Another on-chip auxiliary circuit generates necessary clock and bias for all the blocks. Although the detector is thermally isolated from the substrate of ROIC in theory, the substrate dependent temperature characteristic is obvious. For the low power design, many designs [8,9,10,11] place power-intensive ADC external to ROIC chip. Therefore, ADC-less architecture is also chosen in this work.

### 2.2. IR-Drop Compensation

In large-scale integrated circuits, some long wires exist and exhibit relatively large parasitic resistance. In this design, IR-drop caused by current flowing through long lines has significant influence. When one column of pixels is activated, as illustrated in Figure 1a, the bias currents of all pixels in the column flow through the same blue line, developing an IR-drop along it. For good thermal performance, the width of routing line is often restricted, causing a large line resistance. This results in gradient voltage difference, which cannot be ignored or even be large enough to saturate row readout channel.

For example, the total current is as large as 3.84 mA with nominal 10 μA current source for each pixel. Assuming 100 Ω resistance along the line, large voltage difference as high as 192 mV is presented at the first and last diode output for the same temperature.

To compensate this IR-drop, the reference dummy line as red curve in Figure 1a is introduced to produce varied reference voltage along the vertical direction. Each readout channel offers one extra bias current source for dummy line as well as that for pixel. This configuration guarantees that the dummy line generates the same IR-drop. With differential integrator as first stage in readout channel, the influence of IR-drop is greatly alleviated.

To produce an appropriate drive voltage for dummy line, the blind pixels and feedback mechanism are introduced. Blind pixels have an almost identical structure with normal pixels except that they are shadowed from thermal radiation. One blind pixel row is placed on top of first pixel row, therefore, output voltages from blind pixels can be served as reference voltage. As shown in Figure 1a, the common blind pixel output and top terminal of dummy line are connected to one row channel. The voltage output of this channel is sent to an off-chip amplifier, then low-pass-filtered and buffered to drive the dummy line. With the feedback loop, drive voltage on top of dummy line is forced to be average value of all blind pixel output voltages. As the drive voltage always follows blind pixels when the ambient temperature changes slowly, TEC (Thermo Electric Cooler)-less operation could be achieved.

### 2.3. Residual Non-Uniformity Compensation with Current Splitting DAC

IR-drop can be eliminated totally only if when parasitic resistance and current sources are perfectly matched. This is only partially achieved, and therefore, residual non-uniformities still exist. Besides that, due to process limitation, the variation across IRFPA and mismatch induced input referred offset also can be treated as residual non-uniformity. These non-uniformitis of FPA cause fixed pattern noise (FPN), leading to bad image quality. Only if the residual non-uniformity of IRFPA is further compensated, can better system performance be achieved. By intentionally changing bias current, voltage change can be used to compensate non-uniformity. Therefore, in this design, a variable bias current source with flexible and independent digital control is designed for each pixel.

The classic forward bias current of diode could be written as follows:

(1) $$I=I_{ss}\exp\frac{V}{V_{t}}\;V_{t}=\frac{kT}{q}$$

V${}_{t}$ is thermal voltage (where *k* is Boltzmann constant times, *T* is temperature and *q* is electron charge), $I_{SS}$ is reverse saturation current. Forward voltage changes with bias current as follows,

(2) $$V=V_{t}\ln\frac{I}{I_{ss}}$$

With two times current change, voltage change is about 18 mV at room temperature. Therefore, in this case, with six series connected diode in pixel, residual non-uniformity as large as 108 mV can be effectively compensated.

The change of bias current also has an impact on temperature response coefficient of diode. Take the derivative of voltage to temperature as follows,

(3) $$\frac{dV}{dT}=\frac{k}{q}\ln\frac{I}{I_{ss}}$$

Assume that the forward voltage of a single diode is 0.8 v, and ln(*I*/Iss) is estimated to be 30. Doubling the bias current only leads to a 2.2% change in thermal response coefficient, which is minor and can be compensated easily by a two-point compensation method.

**Figure 2:**
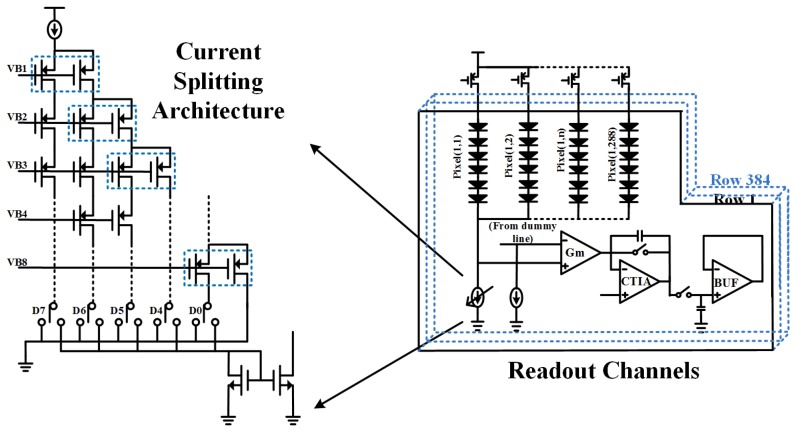
Proposed current splitting architecture-based variable current source.

**Figure 3:**
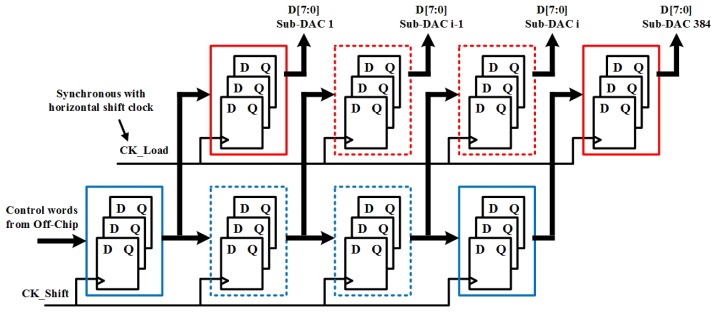
Two bank register arrays for correction word update.

A current splitting architecture is chosen for the variable current source for its compact area and performance [23]. As shown in Figure 2, with equally sized current split transistor in blue box, the nominal 10 μA is divided into eight binary scaled current. These binary currents are combined by using the digital correction word D[7:0] to bias diode pixel. With this design, the current resolution reaches 40 nA. Each pixel has its own correction word for pixel level non-uniformity compensation. Instead of adopting on-chip memory for each pixel as in [22], two banks of register array are designed as shown in Figure 3. When one column of pixel is activated, the top bank register array loads the correction words for this column in parallel and updates synchronously. After this, correction words for next column are serially shifted into register array in the bottom bank. With optimized layout, the proposed current sub-DAC and associated register array occupy a pitch width of 25 μm.

### 2.4. Differential Integrator

**Figure 4:**
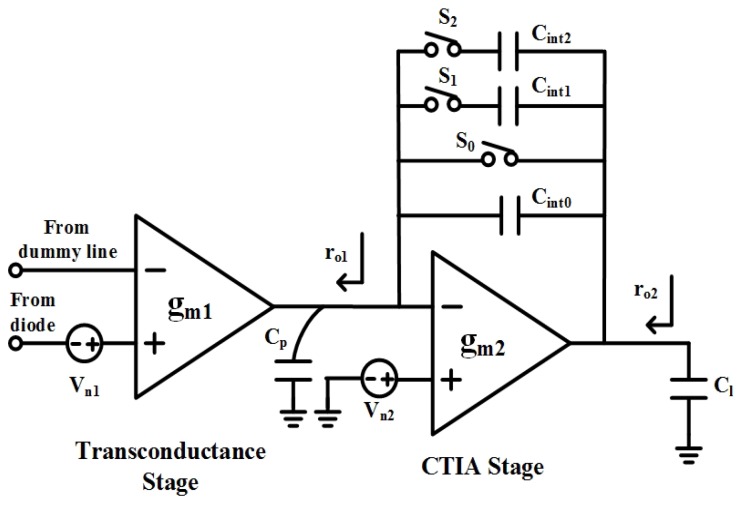
Differential integrator circuit.

Contrary to a microbolometer, voltage signal is present at diode output, but rather than amplifying it with voltage amplifier such as switch capacitor based amplifier directly, a differential integrator-based amplifier is chosen for its low noise performance. The simplified circuit block is shown in Figure 4, it is consists of transconductance stage and capacitor transimpedance amplifier (CTIA). The differential integrator operates in two phases. In the reset phase, reset switch S0 in CTIA closes, the output is forced to a pre-defined voltage. In the amplification phase, the reset switch opens. The weak current converted by the transconductance stage is integrating into capacitor $C_{int}$ continuously. At the end of amplification phase, output voltage is sampled a by later stage. To simplify analysis, amplifiers used in two stages are assumed to be ideal voltage-controlled current source with transconductance of $g_{m1}$/$g_{m2}$, and limited output conductance $g_{o1}$/$g_{o2}$ (Normally, $g_{m1}$/$g_{m2}$ >> $g_{o1}$/$g_{o2}$). The equivalent voltage gain of integrator is approximated as:

(4) $$A_{v0}=\frac{g_{m1}\cdot T_{int}}{C_{int}}$$

where $T_{int}$ and $C_{int}$ are integrating time and integrating capacitor respectively. Voltage gain is proportional to variable integrating time, which is largely defined by frame rate and pixel array size. To accommodate wide dynamic range, two selectable integrating capacitors $C_{int1}$/$C_{int2}$ are connected in parallel with $C_{int0}$.

Integrator noise originates from two operation phases. Assuming only thermal noise of amplifiers is considered, input referred noise power densities are expressed as:

(5) $$V_{n1}^{2}\left(f\right)=4kT\gamma \left(1+\alpha_{1}\right)\frac{1}{g_{m1}}\;V_{n2}^{2}\left(f\right)=4kT\gamma \left(1+\alpha_{2}\right)\frac{1}{g_{m2}}$$

$\alpha_{1}$/$\alpha_{2}$ is the noise factor indicating the noise contribution from transistors other than the input transistor.

Noise in the reset phase causes the output voltage to settle to an uncertain point for later integration. The transfer functions from noise source $V_{n1}$/$V_{n2}$ to output node are:

(6) $$H_{n1,r}\left(f\right)=\frac{V_{o,r}}{V_{n1}}=\frac{g_{m1}/\left(g_{m2}+g_{o1}+g_{o2}\right)}{1+jf/BW_{1,r}}\;BW_{1,r}=\frac{g_{m2}+g_{o1}+g_{o2}}{2\pi (C_{l}+C_{p})}$$

(7) $$H_{n2,r}\left(f\right)=\frac{V_{o,r}}{V_{n2}}=\frac{1}{1+jf/BW_{2,r}}\;BW_{2,r}=\frac{g_{m2}+g_{o1}+g_{o2}}{2\pi (C_{l}+C_{p})}$$

Then we get reset noise power:

(8) $$V_{o,r}^{2}=\int V_{n1}^{2}\cdot H_{n1,r}^{2}\left(f\right)df+\int V_{n2}^{2}\cdot H_{n2,r}^{2}\left(f\right)df\approx \frac{kT}{C_{l}+C_{p}}\left[\frac{g_{m1}}{g_{m2}}\left(1+\alpha_{1}\right)+\left(1+\alpha_{2}\right)\right]$$

In the amplification phase, the transfer function from noise source $V_{n2}$ to output node is changed to:

(9) $$H_{n2,a}\left(f\right)=\frac{V_{o,a}}{V_{n2}}=\frac{1+C_{p}/C_{int}}{1+jf/BW_{2,a}}\;BW_{2,a}\approx \frac{g_{m2}}{2\pi (C_{l}+C_{p}//C_{int})}\frac{C_{int}}{C_{p}+C_{int}}$$

Similaly, $V_{n2}$ induced noise power is as follows (with $C_{int}$ >> $C_{p}$):

(10) $$V_{o2,a}^{2}=\int V_{n2}^{2}\cdot H_{n2,a}^{2}\left(f\right)df\approx \frac{kT}{C_{l}+C_{p}//C_{int}}\frac{C_{int}}{C_{p}+C_{int}}\left(1+\alpha_{2}\right)\approx \frac{kT}{C_{l}+C_{p}}\left(1+\alpha_{2}\right)$$

The derivation of the noise contribution from $V_{n1}$ in amplification phase is not so straightforward. The previous derivation is based on the presumption that the reset/integration time is much longer than the time constant of circuit. In that case, the low frequency voltage gain derived from the transfer function can be applied. For noise source $V_{n1}$ in the amplification phase, this assumption does not hold. For example, in theory, the dc gain can be described as:

(11) $$A_{v,dc}=g_{m1}r_{o1}\cdot g_{m2}r_{o2}$$

But due to the increased time constant in the path and limited integration time, the gain that can be obtained is reduced to $A_{v0}$, and as the noise frequency increases, the equivalent voltage gain decreases. From the intuitive perspective, the integrator can be viewed as an amplifier with dc gain $A_{v0}$ and limited noise bandwidth. In [24], the noise bandwidth is derived as:

(12) $$BW_{1,a}=\frac{1}{2t_{int}}$$

Therefore, noise source $V_{n1}$ introduces an output noise voltage:

(13) $$V_{o1,a}^{2}\approx V_{n1}^{2}\cdot A_{v0}^{2}\cdot BW_{1,2}\approx \frac{2kTg_{m}t_{int}}{C_{int}^{2}}$$

Adding all the noise power together, we can get input inferred noise:

(14) $$V_{n}=\frac{\sqrt{V_{o,r}^{2}+V_{o1,a}^{2}+V_{o2,a}^{2}}}{A_{v0}}=\sqrt{\frac{kT}{C_{l}+C_{p}}\left[\frac{g_{m1}}{g_{m2}}\left(1+\alpha_{1}\right)+2\left(1+\alpha_{2}\right)\right]\frac{1}{A_{v0}^{2}}+\frac{2kT}{g_{m1}t_{int}}}$$

**Figure 5:**
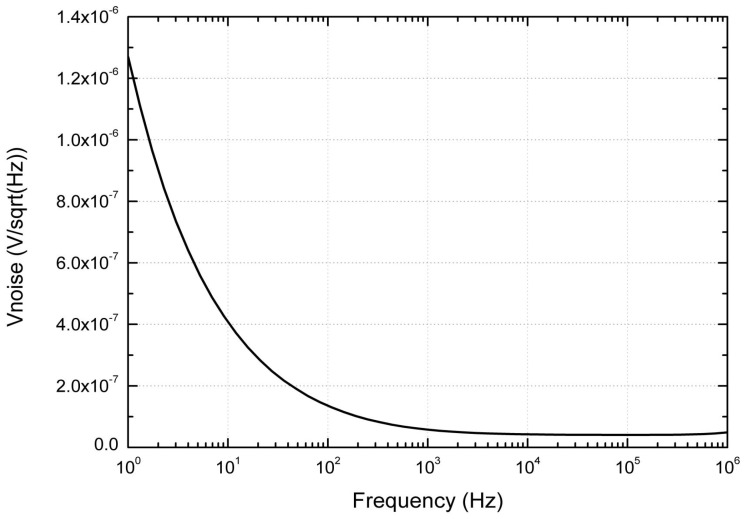
Differential integrator circuit.

Based on this equation, some design choices are made to achieve low noise performance, such as telescope cascode architecture for amplifier to lower $\alpha_{1}$/$\alpha_{2}$, optimized integrating time and large integrating capacitor. Figure 5 presents the simulated noise; the input inferred noise is less than 4.7 μV within 50 KHz bandwidth.

### 2.5. Low Power Row Buffer Design

Power consumption is always one major concern in IC design, especially in infrared sensor application. Large power consumption can cause a self-heating phenomenon, which leads to temperature change in the detector. Although the substrate temperature of detector can be stabilized by using thermo electric cooler, it is still necessary to reduce power consumption.

**Figure 6:**
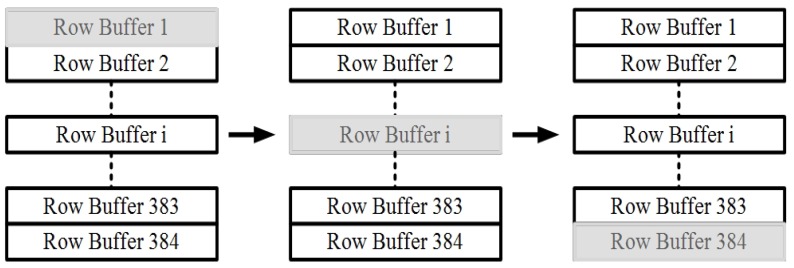
Differential integrator circuit.

The row buffer is the major source of power consumption, as it needs to drive large parasitic capacitance on a shared bus in the multiplexer, and stabilize within one readout clock. By analyzing readout operation as shown in Figure 6, it is clear that there is plenty of room for power reduction. As one column of pixels is initiating the readout process, all the row buffers are working continuously, but only one channel is multiplexed to the output. It can be inferred that it is possible to turn off the row buffer in other channels.

**Figure 7:**
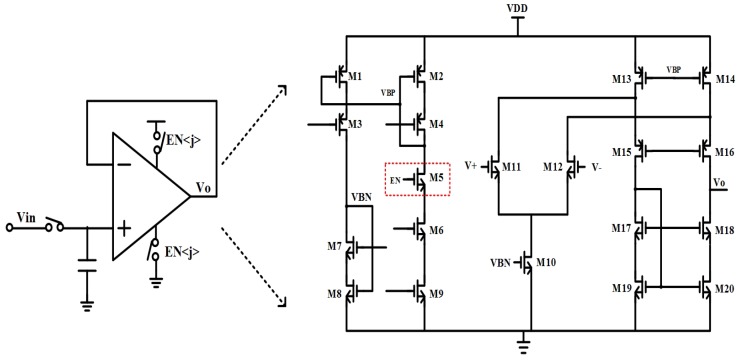
Proposed pulse powered row buffer.

Therefore, a row buffer with pulse-powered capability is proposed in this work. As shown in Figure 7, the core architecture of the row buffer is one stage fold-cascoded amplifier with differential NMOS input transistors. The bias network for amplifier is used for power control purposes. When EN is high, the row buffer works in normal mode. If EN is low, VBP turns high, and the current of the buffer is cut down.

Taking recovery time and robust operation into consideration, the buffer is turned on one clock before the row is actually multiplexed to output. Also, the buffer is turned off one clock after the row output is completed. By this method, only three row buffers work at the same time, and the extra power consumption is acceptable.

## 3. Experiment Results

**Figure 8:**
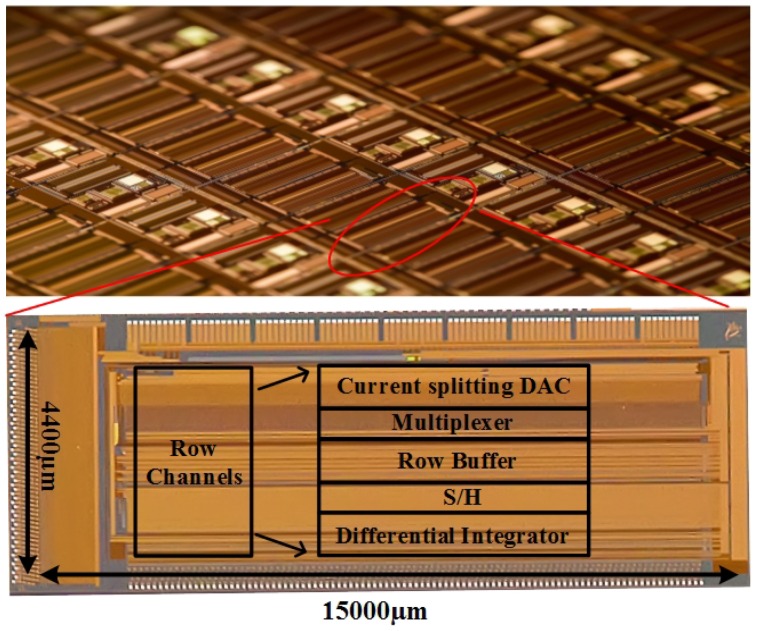
Microphotograph of designed ROIC.

The front-end ROIC for the 384 × 288 diode-based infrared sensor with on-chip non-uniformity compensation technique is designed and fabricated with 0.35-μm 2-poly and 3-metal CMOS process. The microphotograph of the ROIC sample is shown in Figure 8, the core area is 4.4 mm × 15 mm, and power consumption of the whole chip is about 180 mW.

**Figure 9:**
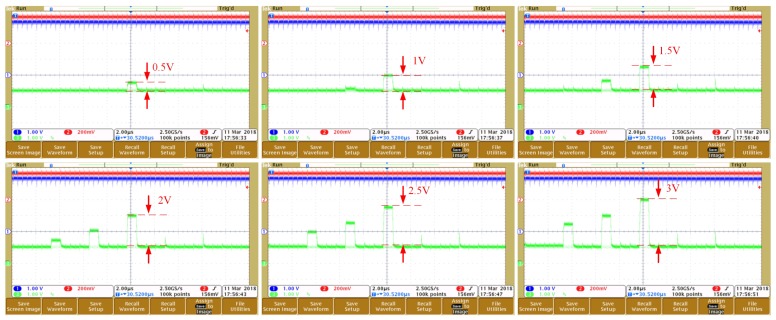
ROIC output voltage with increased temperature.

**Figure 10:**
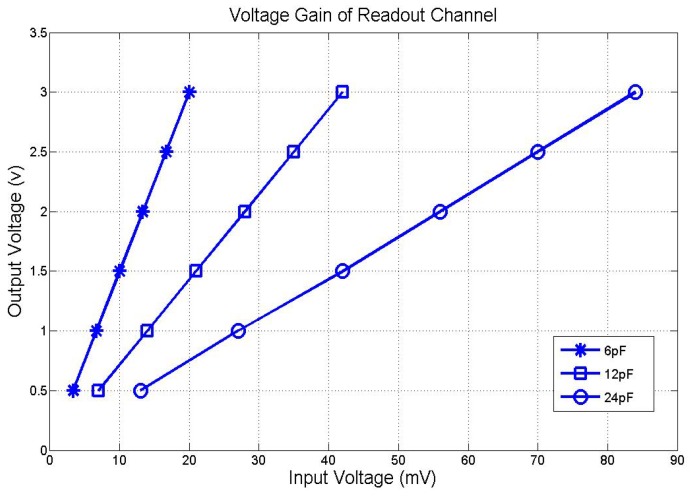
Measured voltage gain of proposed ROIC.

Due to a limited available pad, in testing mode, only 16 row channels are connected to the diode array by bonding wires for rapid evaluation of the ROIC. An FPGA is used as DSP for the DAC array and an oscilloscope is used to capture the output voltages of ROIC. As shown in Figure 9, as the temperature increases, the output voltage range can extend from 0.5 v to 3 v. The accurate voltage gains for different integrating capacitors are drawn in Figure 10. With 100 μS integration time, voltage gains with 6/12/24 pF integrating capacitor are 150/75/37.5, respectively.

**Figure 11:**
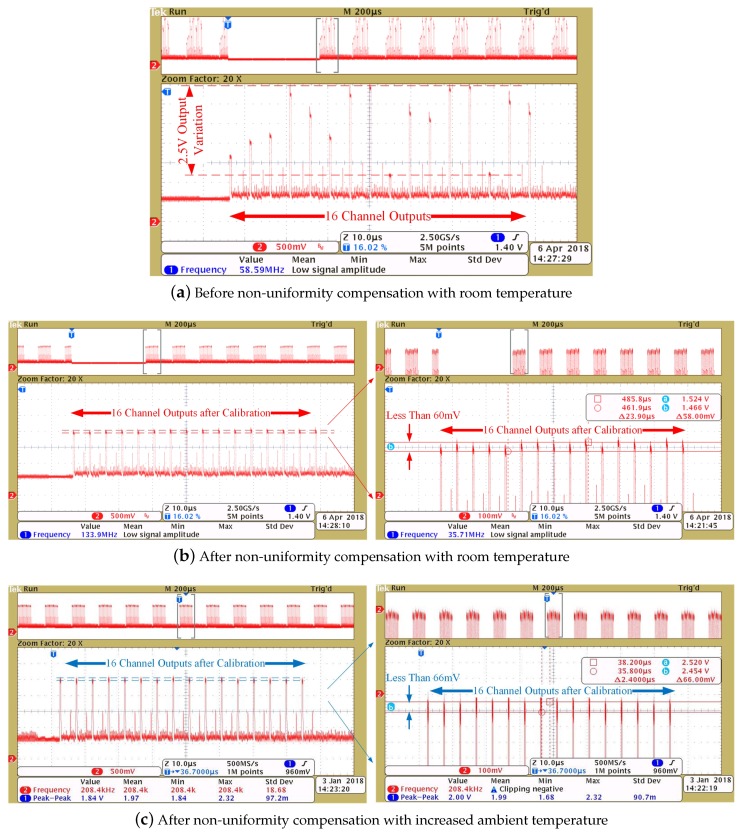
Output voltage variations before and after non-uniformity compensation.

To evaluate the calibration performance of the proposed ROIC, the measurements are done as follows. First, at room temperature, all the pixels are biased by the same current, and the outputs are shown in Figure 11a. Then, the current splitting DACs in these channels are adjusted according to the corresponding outputs to compensate for the non-uniformity. The outputs after calibration are shown in Figure 11b. Both of the outputs are sampled with the same input signal under the same condition. The output variations of the ROIC are about 2.5 V and 60 mV before and after calibration, respectively, which validates a more than 97% non-uniformity improvement. By increasing the ambient temperature, the output voltage exhibits a litter larger variation as much as 66 mV without re-calibration, as depicted in Figure 11c. This tolerable discrepancy is caused by the minor spreads in the temperature response of diode detector and the voltage gain of each readout channel.

## 4. Conclusions

In this paper, a new ROIC for diode IRFPA with the nonuniformity compensation technique is presented. A reference dummy wire is utilized to compensate for voltage drop in focal plane array. A novel current splitting DAC array is proposed to compensate for residual non-uniformity in both detector array and ROIC. With the help of off-chip ADC and digital microprocessor, the non-uniformity of the diode detector array and the mismatch of current source can be stored and then calibrated. An ROIC for the 384 × 288 diode detector array is fabricated with a 0.35 μm CMOS process, the chip occupies an area of 4.4 mm × 15 mm, and power consumption is 180 mW. With IR-drop calibration and current splitting DAC compensation, the output voltage variations are 2.5 V and 60 mV before and after compensation, respectively. The non-uniformity compensation technique has been proven very effective with 97% reduction.
